# MRI-Based Radiomics Reveals Cannabinoid-Associated Tumor Phenotypes in a Murine Breast Cancer Model

**DOI:** 10.3390/molecules31071154

**Published:** 2026-03-31

**Authors:** Ioana Creanga-Murariu, Cosmin-Vasilica Pricope, Mitica Ciorpac, Debbie Anaby, Kfir Cohen, Cristina-Mariana Uritu, Andrei Szilagyi, Raluca-Maria Gogu, Wael Jalloul, Adriana-Elena Anita, Dragos-Constantin Anita, Radu-Andrei Baisan, Teodora Alexa-Stratulat, Bogdan-Ionel Tamba

**Affiliations:** 1 Advanced Research and Development Center for Experimental Medicine “Prof. Ostin C. Mungiu” (CEMEX), Grigore T. Popa University of Medicine and Pharmacy, 700115 Iasi, Romania; 2 Medical Oncology-Radiotherapy Department, Grigore T. Popa University of Medicine and Pharmacy, 700115 Iasi, Romania; 3 Department of Medical Oncology, Regional Institute of Oncology, 700483 Iasi, Romania; 4 Radiology Department, “Sfantul Spiridon” Hospital, 700111 Iasi, Romania; 5 Department of Diagnostic Imaging, Sheba Medical Center, Tel-Hashomer, Ramat Gan 52621, Israel; 6 Department of Biophysics and Medical Physics-Nuclear Medicine, Grigore T. Popa University of Medicine and Pharmacy, 700489 Iasi, Romania; 7 Regional Center of Advanced Research for Emerging Diseases, Zoonoses and Food Safety, Faculty of Veterinary Medicine, Iași University of Life Sciences (IULS), 8 Mihail Sadoveanu Alley, 700489 Iasi, Romania; 8 Faculty of Veterinary Medicine, “Ion Ionescu de la Brad” Iasi University of Life Sciences, 700489 Iasi, Romania

**Keywords:** CBD, THC, cannabinoids, radiomics, breast cancer

## Abstract

Introduction and Aim: Assessment of antitumor activity in preclinical models remains challenging when relying solely on conventional size-based imaging, particularly for complex agents such as cannabinoids, whose biological effects may not translate into early volumetric tumor changes. Cannabinoid formulations, including the synthetic cannabinoid JWH-182, Cannabixir^®^ Medium dried flowers, and Cannabixir^®^ THC full extract, exhibit diverse and potentially subtle effects on tumor biology. Radiomics enables high-throughput extraction of quantitative imaging features that capture intratumoral heterogeneity beyond gross tumor volume. The primary aim of this study was to evaluate the utility of MRI-based radiomics as a sensitive tool for detecting cannabinoid-associated tumor phenotypic modulation in a preclinical breast cancer model. Methods: Orthotopic breast tumors were induced in mice using the 4T1 cell line. Animals received cannabinoid formulations in combination with chemotherapy according to a predefined protocol. Tumor burden was assessed at baseline and post-treatment using ultrasonography and whole-body MRI to calculate tumor doubling time. T1- and T2-weighted MRI datasets were segmented and analyzed using radiomics to extract morphometric and signal-based features. Results: Conventional imaging revealed no significant differences in tumor doubling time between most cannabinoid-treated groups and controls, except for accelerated growth in animals treated with Cannabixir^®^ THC full extract. In contrast, radiomics identified distinct, compound-specific tumor phenotypes, including structural features consistent with reduced aggressiveness, in JWH-182-treated tumors, despite similar volumetric growth patterns. Conclusion: MRI-based radiomics sensitively captures cannabinoid-associated tumor phenotype alterations beyond volumetric assessment, supporting its value as a pharmaco-imaging tool for characterizing treatment-related tumor biology in preclinical oncology.

## 1. Introduction

Cancer is the second leading cause of death and morbidity on a global scale, accounting for around 9.7 million deaths each year [1]. It is a health, social, and humanitarian issue that causes significant suffering and is responsible for the highest costs in healthcare systems for disease management [2,3]. Chemotherapy is an effective tool for slowing the progression of cancer; nevertheless, it comes with a tendency to resistance and a variety of side effects [4]. Thus, there is always a constant need to develop alternative or synergistic anticancer drugs with a good safety profile. One interesting strategy to develop effective antitumoral agents is to study anticancer agents derived from natural sources proven to be effective for cancer prevention and therapeutics, such as vinka-alkaloids, polyphenols or taxanes [5].

Recent discoveries identified the benefits of Cannabis sativa and its synthetic analogues towards the modulation of cancer, either directly in tumor homeostasis or in the morphology and function of normal processes modified by cancer. Cannabis contains over 450 compounds, with at least 100 fat-soluble molecules called phytocannabinoids, which can be found in the resin produced by cannabis plants. The best known are tetrahydrocannabinol (THC) and cannabidiol (CBD), which modulate the endocannabinoid system that consists of cannabinoid receptors (CBRs), enzyme signaling molecules, etc. [6]. Synthetic cannabinoids are laboratory-synthesized drugs, functionally similar to phytocannabinoids, but ten times more potent than natural compounds, presenting complete agonist features and higher affinity for cannabinoid receptors [7].

One important aspect is that cannabinoids have been reported to produce antitumor effects in several preclinical cancer models by tackling different stages of cancer progression, such as uncontrolled cancer cell proliferation, apoptosis, angiogenesis and metastasis [8]. Further studies showed that cannabinoids could be a potential combination partner for established chemotherapeutic agents or other therapeutic interventions in cancer treatment [9]. Fewer studies, however, raised the issue that the antitumor activity of cannabinoids is highly dependent on the type of receptor agonist, target tumoral tissue, route of administration, doses and duration of the overall exposure, and could, in fact, shift the balance towards an increase in tumor growth and aggressiveness [10]. Although the antitumor properties of cannabinoids are merely explored in the preclinical setting, two randomized controlled trials have already been conducted. The phase Ib trial conducted by Twelves et al. and the phase 2 trial performed by Schloss et al. investigated the antitumor activity of Nabiximols and THC products, respectively, against placebo in the case of advanced glioblastoma patients. They found an increased survival rate in the cannabinoid group (83% vs. 44%) and 61% reduction in the initial tumor mass [11,12].

Although cannabinoids are promising candidates to be used in the oncology setting, new synthetic compounds and novel plant strains with distinct pharmacokinetic and pharmacodynamic properties continue to be developed; needless to say, research being conducted in this area is still considered to be in the “drug discovery” phase [13]. Before new cannabinoids enter the clinical study phase, mainly when a chronic pathology is involved, it is critical to identify reliable biomarkers that validate the medication’s efficacy and safety [14].

The use of imaging biomarkers for drug evaluation, a field known as “pharmaco-imaging”, can aid in lowering research needs, resulting in faster results with good statistical power [15]. When discussing the efficacy of herbal medications in animal and human studies, morbidity evaluation can sometimes be subjective, making an accurate evaluation of the results difficult. In contrast, imaging biomarkers can be more objectively, quickly, and easily evaluated. The resulting images may reveal subtle changes that could be missed using conventional clinical assessment [16]. Furthermore, animal studies often involve the sacrifice of animals for the histopathology analysis of a drug’s effects, whereas monitoring the drug’s effectiveness by imaging techniques is a tool to be used several times on the same animal, allowing an ethical approach to preclinical research [15].

As imaging techniques play a vital role in managing cancer patients, improved techniques with better image quality and novel analysis methods are always developing to provide highly personalized and detailed information. New trends place radiomics, machine learning and artificial intelligence (AI) at the top of the imaging biomarker pyramid, given that they are rapidly expanding fields that show great promise for enhancing medical image interpretation [17]. Radiomics is an innovative concept based on extracting an extensive set of quantitative features from images to identify patterns, correlations and hidden information that cannot be detected with the naked eye or with traditional image analysis methods [18]. It is based on the idea that each pixel in a medical image contains essential information about tissues and structures within the body. It provides detailed and subtle information that can have significant implications for diagnosis, prognosis and tracking disease progression [16,18]. This approach applies to images generated from computed tomography (CT), magnetic resonance imaging (MRI), ultrasound and even positron emission tomography (PET) [19]. Radiomics can play a valuable role in oncological drug discovery due to its ability to generate imaging biomarkers, which can serve as non-invasive indicators for tissue characterization and, consequently, a more precise assessment of tumors’ dynamics in response to treatment [20,21]. It has benefits outside preclinical research, with great value for translation to the human population. Physicians can develop treatment plans tailored to patients’ needs by analyzing clinical, genetic, and radiometric information, which further significantly improves therapeutic outcomes and patients’ overall quality of life [16,17,22].

The present study is motivated by the need for new sensitive tests to emphasize the true benefits of complex medicinal products, the strong need for new, efficient, and non-toxic treatments in cancer and the attractive roles of cannabinoids as antitumor agents. Therefore, our aim is to analyze the sensitivity of radiomics as a tool to predict tumor response, compared to conventional ultrasonography and MRI scans, by using imaging biomarkers such as tumor radiomic signatures. For this, we screened one synthetic cannabinoid, JWH-182 and two phytocannabinoids, Cannabixir^®^ Medium dried flowers (NC1) and Cannabixir^®^ THC full extract (NC2), for their potential to induce antitumoral modifications in a breast cancer murine model, assessed with ultrasonography and MRI, using a radiomic approach.

## 2. Results

### 2.1. Visual Tumor Characterization Using 2D Ultrasonography and Axial and 3D Reconstructed MRI Images

**Figure 1:**
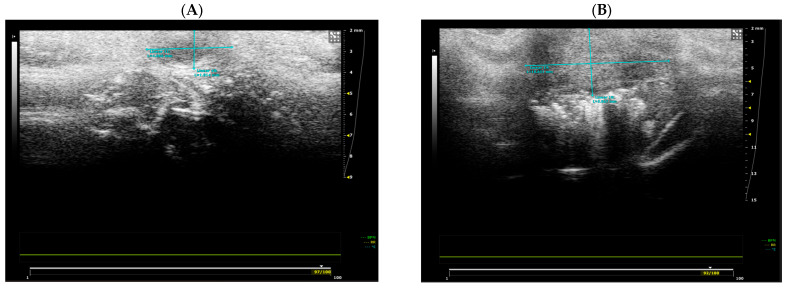
Comparative aspects of ultrasonography-assessed tumors at baseline (**A**) and the end of the treatment period (**B**). Tumor dimensions were measured using the ultrasound system caliper tool (blue lines), indicating the anteroposterior and craniocaudal diameters (mm). Tumors appear as non-homogeneous structures with irregular extensions in the mammary gland.

When analyzed with ultrasonography, the tumors were hypoechogenic with a non-homogeneous internal structure and lobulated contour, associated with a hyperechogenic peripheral halo and the appearance of a reticulated infiltrate of peritumoral fat (Figure 1). The tumors were superficially located, just underneath the skin, sending irregular extensions deep into the mammary gland.

**Figure 2:**
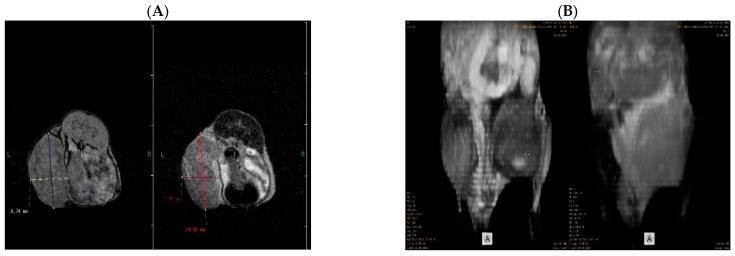
Comparative aspects between axial images (**A**) and 3D reconstruction (**B**) of the breast tumor in both T1 (left) and T2 (right) at the end of the experiment. These images highlight the position and burden of the tumor and its overall relationship with the surrounding tissues. Axial images offer greater information regarding the tumor margins and consistency, whereas 3D reconstruction is more accurate in emphasizing the tumor’s overall compressive aspect and relationship with neighboring anatomical structures. Acquisition of whole-body MRI images was performed using 1T PET/MRI (nanoScan^®^, Mediso Medical Imaging Systems, Budapest, Hungary).

Axial and 3D reconstructed images provided different aspects regarding tumor structure and the relationship with the surrounding tissues, which, in fact, provided complementary information. Figure 2A (native axial section) and Figure 2B (3D reconstruction) show the overall appearance of the breast tumors scanned at the end of the experiment. The tumors appear as heterogeneous masses, localized on the lower right abdominal wall, corresponding to the initial inoculation site of the 4T1 cells.

In case of the 3D reconstructed images, features such as abdominal viscera and the musculoskeletal structures of the right hip are clearly observable as distorted due to the tumor mass, which compresses the normal anatomical structures, suggesting, at a glance, the installation of pain and functional impairment, observable in all the animals, regardless of the treatment. However, we could not observe this feature with the axial images, suggesting that 3D reconstruction provides the examiner with a more suggestive overview of the overall compressive effect of the tumor. This aspect can be seen in Figure 2A,B.

Standard axial images bring valuable information regarding the overall aspect of the tumor. We observed that the tumor masses of animals in all treatment groups presented a high grade of heterogeneity, mainly due to the presence of areas of necrosis and edema. Compared to the 3D reconstruction, tumor margins are better marked on axial T2 slices, which is facilitated by the peritumoral edema (in hypersignal), which outlines the tumor and creates an optimal contrast.

### 2.2. Treatment Efficacy Using Conventional Ultrasonography and Standard and VOI-Segmented MRI Images

**Figure 3:**
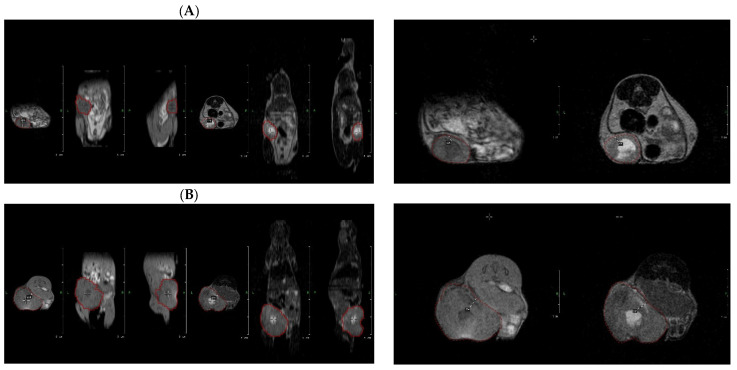
Tumor tissue segmentation and VOI definition at baseline (**A**) and after treatment (**B**). VOI definition of the tumor at the baseline imaging staging (day 8) in T1; red highlight (transversal, craniocaudal and anteroposterior aspects); and T2; red highlight (transversal, craniocaudal and anteroposterior aspects). VOI definition of the tumor at the end of the experiment (day 26) in T1; red highlight (transversal, craniocaudal and anteroposterior aspects); and T2; red highlight (transversal, craniocaudal and anteroposterior aspects).

Marked changes in tumor volume and internal composition were observed between baseline and the end of the treatment period across all experimental groups, irrespective of the administered compound or imaging modality. All animals exhibited a pronounced increase in tumor size accompanied by progressive distortion and infiltration of adjacent tissues. Representative ultrasonographic and MRI findings are illustrated in Figure 1 and Figure 3, respectively, highlighting morphological features that were consistently present across treatment groups.

Serial imaging demonstrated a clear enlargement of the primary tumor relative to baseline measurements, associated with a substantial mass effect on surrounding abdominal and musculoskeletal structures. Notably, tumors remained contiguous with the peritoneal layers, without evidence of a discernible cleavage plane, a feature suggestive of locally invasive growth. On MRI, the centrally located necrotic component, characterized by a hyperintense signal in T2-weighted images, persisted throughout the study and expanded in parallel with overall tumor growth, accompanied by progressive peritumoral edema.

In addition, rapid tumor expansion resulted in surface disruption, with focal peripheral ulceration becoming apparent at the final imaging time point. This finding was visualized as a localized depression along the posterior tumor margin, displaying subtle hyperintensity on T1-weighted sequences, consistent with superficial tissue breakdown secondary to tumor overgrowth.

Tumor growth kinetics, expressed as tumor volume doubling time, were evaluated across treatment groups using US as well as conventional and VOI-segmented T1- and T2-weighted MRI datasets. No statistically significant differences in doubling time were detected between cannabinoid-treated groups and controls, regardless of the imaging modality employed.

**Figure 4:**
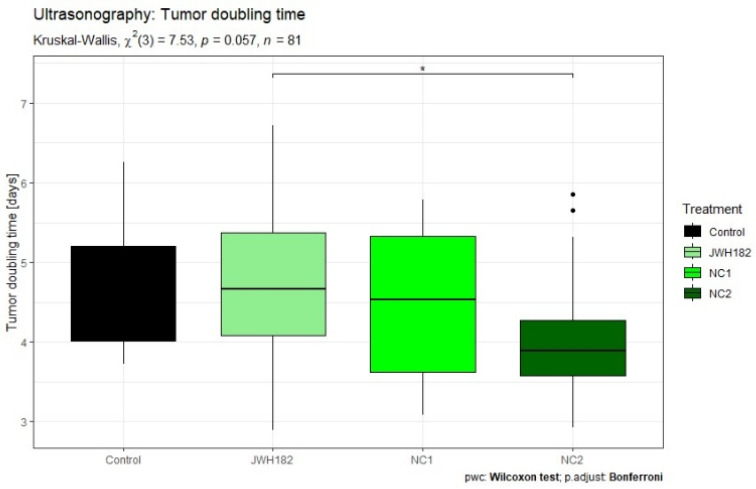
Ultrasonography measured tumors of animals treated with either JWH-182 (*n* = 29), NC1 (*n* = 23), or NC2 (*n* = 23), and finally controls (*n* = 6). Three tumor dimensions were recorded (anteroposterior, transversal and craniocaudal) and tumor volume was calculated as mentioned. Results are expressed as doubling time (DT), calculated to estimate the time required for the tumor to double in volume. * Indicates a significant pairwise difference between JWH182 and NC2 based on post hoc Wilcoxon rank-sum testing with Bonferroni correction.

**Figure 5:**
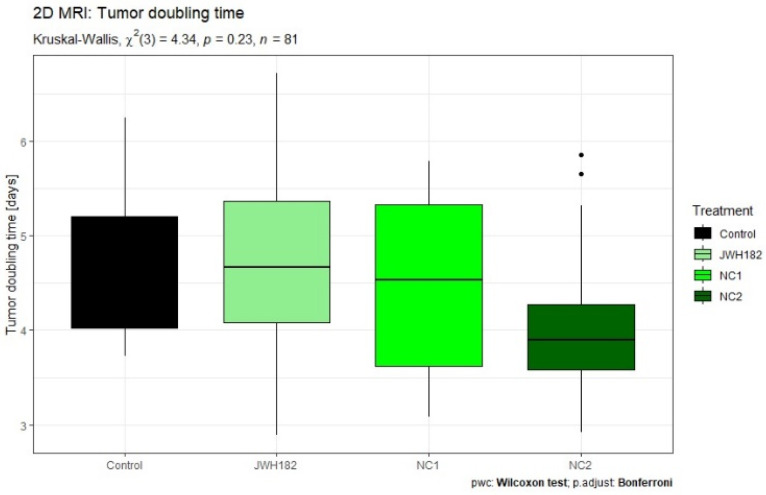
Standard MRI measured tumors of animals treated with either JWH-182 (*n* = 29), NC1 (*n* = 23), or NC2 (*n* = 23), and finally controls (*n* = 6). Three tumor dimensions were recorded (anteroposterior, transversal and craniocaudal) and tumor volume was calculated as mentioned. Results are expressed as doubling time (DT), calculated to estimate the time required for the tumor to double in volume.

Across most experimental groups, including controls as well as animals treated with JWH-182 or NC1, tumors exhibited comparable growth behavior, with volume doubling occurring approximately every four to five days. In contrast, a consistent trend toward accelerated tumor growth was observed in animals receiving NC2. In this group, tumors reached a twofold increase in volume within a shorter interval of approximately three to four days, a pattern that was reproducible across US measurements and standard T2-weighted MRI assessments, as illustrated in Figure 4 and Figure 5. While the overall comparison of tumor doubling time among groups did not reach statistical significance according to the Kruskal–Wallis test (*p* = 0.0567), exploratory post hoc Wilcoxon rank-sum tests with Bonferroni correction revealed a significant difference between JWH-182 and NC2 (raw *p* = 0.004; adjusted *p* = 0.025), whereas no other pairwise comparisons were significant.

Volumetric tumor segmentation was conducted on both T1- and T2-weighted MRI datasets to account for sequence-dependent differences in signal intensity and tissue contrast. This approach enabled a comprehensive three-dimensional characterization of tumor architecture, allowing visualization of intratumoral heterogeneity, including regions consistent with hemorrhage, necrosis, and peritumoral edema, features observed across all experimental groups.

**Figure 6:**
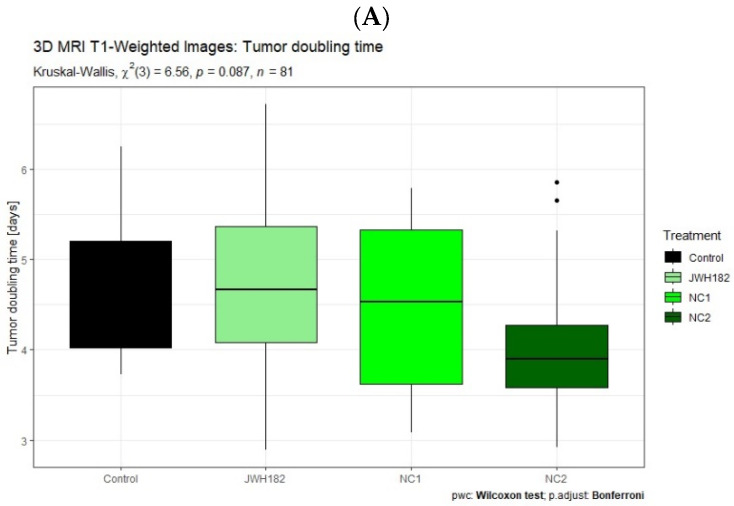

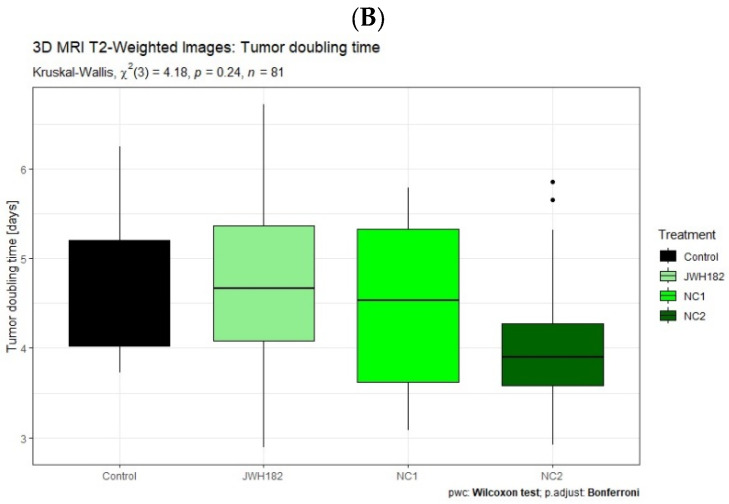
VOI-segmented MRI T1 (**A**) and T2 (**B**) tumors of animals treated with either JWH-182 (*n* = 29), NC1 (*n* = 23), or NC2 (*n* = 23), and finally controls (*n* = 6). Automatically calculated after manual segmentation and obtaining the tumor volume of interest. Results are expressed as doubling time (DT), calculated to estimate the time required for the tumor to double in volume.

When tumor growth kinetics derived from VOI-based segmentation were compared between imaging sequences, no statistically significant differences in tumor doubling time were identified in the control, JWH-182, or NC1 groups. In contrast, tumors in the NC2-treated group exhibited a consistent trend toward shorter doubling times in both T1- and T2-weighted images, suggesting a more aggressive growth pattern. This sequence-independent trend is illustrated in Figure 6 and mirrors the findings obtained from non-segmented imaging assessments.

### 2.3. Predictability of Radiomics as a Tool for Detecting Cannabinoid-Associated Tumor Phenotypic Modulation

Alterations in MRI-derived radiomic features between baseline and post-treatment imaging were systematically evaluated for both control and cannabinoid-treated groups using T1- and T2-weighted datasets. In the control group, none of the extracted radiomic features demonstrated statistically significant temporal changes. In contrast, tumors exposed to cannabinoid treatment exhibited substantial radiomic modulation, with more than 55% of the analyzed features showing significant differences between baseline and end-of-treatment values.

Across all cannabinoid-treated groups, first-order and morphometric features, including tumor volume and maximum diameter, increased significantly over time, reflecting progressive tumor growth irrespective of treatment type, as shown in Table 1. Beyond size-related metrics, multiple higher-order texture features displayed consistent modulation, including entropy, homogeneity, size variability, skewness, coarseness, and busyness, as well as zone- and run-length-based descriptors (short- and long-zone emphasis, short- and long-run emphasis). These changes indicate pronounced alterations in intratumoral heterogeneity and spatial organization that were not captured by conventional volumetric assessment.

**Figure 7:**
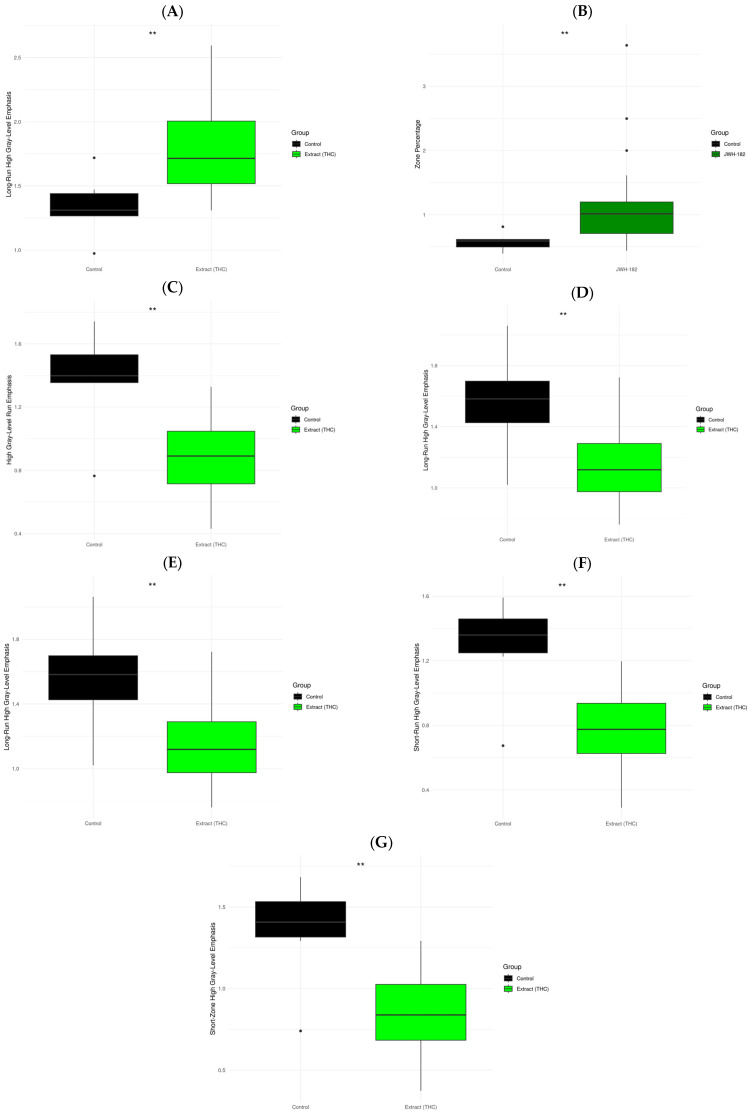
Ratio values of radiomic features that showed significant differences between the control group and one of the treatment groups from the T1 images (**A**,**B**) and the T2 images (**C**–**G**). Data are mathematically expressed as the ratio of post-treatment to baseline feature values to account for the repeated-measures structure. Statistical comparisons were performed using exact non-parametric permutation tests based on *t*-test statistics, with false discovery rate (FDR) correction for multiple comparisons. * *p* < 0.05, ** *p* < 0.01.

To determine whether radiomic modulation was treatment-specific, feature value ratios were compared between each cannabinoid group and the control group. Figure 7 summarizes the radiomic features that remained statistically significant after false discovery rate (FDR) correction for multiple comparisons in both T1- and T2-weighted images. The most extensive differences were observed between control tumors and those treated with NC2, involving multiple radiomic descriptors. Notably, this pronounced radiomic divergence occurred in parallel with accelerated volumetric tumor growth, suggesting that radiomics captures treatment-associated tumor phenotype alterations rather than therapeutic efficacy. By comparison, a single feature (zone percentage) showed a significant difference between the control group and tumors treated with JWH-182, while no radiomic features survived FDR correction in comparisons involving the NC1 group.

### 2.4. Histopathological Findings

**Figure 8:**
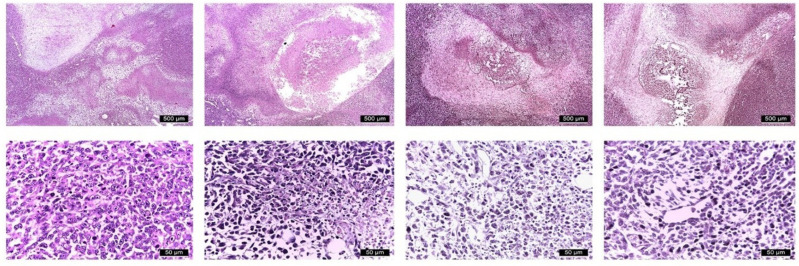
Representative H&E-stained histological sections of primary tumors. Columns correspond to treatment groups (PTX, JWH-182, NC1, NC2). The first row shows the overall tumor architecture, scale bar: 500 µm; the second row presents higher-magnification views, scale bar: 50 µm.

Histopathological examination confirmed the aggressive phenotype of the 4T1 mammary tumors across all experimental groups (Figure 8). The tumors were composed of poorly differentiated anaplastic malignant cells with marked cytological atypia, including nuclear pleomorphism, hyperchromatism, prominent nucleoli, and increased mitotic activity. Peripheral tumor regions showed dense tumor cell proliferation associated with fibroblasts, collagen deposition, and inflammatory cell infiltration consisting of macrophages, neutrophils, eosinophils, and lymphocytes. These morphological characteristics were largely consistent across treatment groups and reflect the highly invasive nature of the 4T1 model.

In contrast, the central tumor areas displayed reduced cellular density with extracellular matrix accumulation, numerous apoptotic bodies, and extensive regions of necrosis, which were observed in approximately one-third of the animals across experimental groups. Necrotic regions consisted of disorganized cellular debris with indistinct cellular boundaries, pyknotic nuclei, and eosinophilic cytoplasmic remnants, consistent with high-grade malignant lesions, similar in all treatment lines.

## 3. Discussion

Accurately assessing anticancer treatment efficacy remains challenging due to the intrinsic biological heterogeneity of tumors, variability in treatment response, and the frequent dissociation between molecular or microstructural changes and macroscopic tumor size. Conventional imaging-based metrics, while indispensable in clinical practice, primarily capture late or cumulative effects of therapy and may therefore overlook early, subtle, or non-volumetric treatment-induced alterations.

In this context, the present study was designed to evaluate whether radiomics-based image analysis can serve as a sensitive pharmaco-imaging tool capable of detecting treatment-associated tumor phenotypic modulation produced by cannabinoids that is not apparent using standard ultrasonography or MRI-derived volumetric measurements.

Our findings demonstrate that radiomic analysis of MRI data identifies exposure-dependent tumor signatures that remain undetected by conventional imaging metrics. Across all experimental groups, including controls, primary tumor volume increased substantially over time, and tumor doubling time did not differ significantly between most cannabinoid-treated groups and controls. These results, obtained consistently across ultrasonography and conventional MRI, indicate that volumetric growth kinetics alone are insufficient to capture treatment-related biological modulation in this aggressive 4T1 model. While MRI provides superior anatomical detail and enables radiomic analysis, ultrasonography offers a faster and more accessible method for routine monitoring of tumor growth in preclinical models. Importantly, radiomics revealed distinct and compound-specific alterations in tumor texture and spatial organization despite similar growth trajectories, directly supporting our central hypothesis regarding the sensitivity of radiomics to non-volumetric tumor changes.

Radiomics extends beyond traditional radiological assessment by quantifying tumor heterogeneity, texture, and internal organization, features that reflect underlying cellular density, necrosis, edema, angiogenesis, and stromal remodeling. Rather than relying on a single scalar parameter or subjective visual interpretation, radiomics effectively functions as a “virtual biopsy,” capturing multidimensional information from routinely acquired imaging datasets [23,24].

Previous clinical studies have demonstrated the predictive value of radiomics in breast cancer, including its ability to anticipate pathological complete response and treatment sensitivity using PET/MRI or multiparametric MRI feature sets [22,24]. The present study translates these principles into a preclinical setting and extends them to the evaluation of pharmacological exposures with complex, heterogeneous, and context-dependent biological effects.

In the present analysis, no radiomic features showed significant temporal modulation in the control group after correction for multiple comparisons, indicating relative stability of imaging phenotypes in the absence of cannabinoid exposure. In contrast, more than half of the radiomic features extracted from cannabinoid-treated tumors exhibited significant changes between baseline and post-treatment imaging. These changes involved not only morphometric parameters but also higher-order texture features, including entropy, homogeneity, run-length-, and zone-based descriptors, underscoring substantial alterations in intratumoral architecture and spatial complexity [24]. Notably, these radiomic modulations occurred independently of tumor volume expansion, reinforcing the notion that radiomics captures biologically relevant information beyond size alone.

When treatment-specific effects were examined, radiomic signatures proved to be highly compound-dependent. Tumors exposed to the synthetic cannabinoid JWH-182 demonstrated a significant change in the “zone percentage” feature in T1-weighted images, a metric reflecting the relative dominance of homogeneous regions within the tumor volume. A shift toward reduced intratumoral heterogeneity has been associated with lower-grade malignancy and improved differentiation in multiple oncologic contexts [25]. Within the limitations of imaging-based inference, this finding suggests that JWH-182 may induce partial structural normalization within the tumor microenvironment rather than overt growth inhibition. This interpretation is consistent with previously reported antitumor and chemosensitizing effects of synthetic cannabinoids with preferential CB2 receptor activity, including those described in our recent study, where JWH-182 reduced metastatic burden and modulated tumor-associated biological pathways [26].

In contrast, NC2-treated tumors exhibited the most extensive radiomic divergence from controls, characterized by significant alterations in multiple second-order texture features across both T1- and T2-weighted images. Several of these changes, including increased long-run gray-level emphasis and reduced post-treatment feature ratios, have been associated with heightened heterogeneity, hypoxia, and aggressive tumor phenotypes [27]. Importantly, these radiomic findings occurred in parallel with a trend toward accelerated tumor growth observed using conventional imaging, indicating that radiomics sensitively captures phenotypic shifts without implying therapeutic benefit. This pattern is consistent with reports of protumoral or context-dependent effects associated with THC-dominant formulations in certain breast cancer models [26].

By contrast, no radiomic features survived false discovery rate correction in the NC1-treated group, suggesting a comparatively neutral impact on primary tumor architecture within the temporal and methodological limits of this study. This observation does not contradict previously reported antitumor or antimetastatic effects of phytocannabinoids but instead highlights the context-specific nature of cannabinoid action and the limitations of primary tumor imaging as a sole efficacy endpoint [28]. This finding aligns with our prior study, where NC1 demonstrated reduced metastatic spread when assessed through histopathological analysis of target organs, while imaging-based evaluation did not reveal significant antitumor effects on the primary tumor [26]. Cannabinoids may exert clinically relevant effects through immune modulation, suppression of metastatic dissemination, or symptom palliation, mechanisms that are not necessarily reflected in early changes in primary tumor growth or structure [29].

Such structural complexity is known to influence imaging texture patterns. For example, increased entropy has been associated with greater intratumoral heterogeneity and necrosis [30], whereas homogeneity reflects more uniform tissue architecture and cellular organization [28]. Similarly, run-length and zone-length texture metrics are influenced by spatial variations in cellular density, stromal composition, and extracellular matrix organization. Therefore, the radiomic signatures identified in this study likely reflect underlying heterogeneity rather than tumor size alone, supporting the concept that radiomics can capture biologically relevant tumor phenotypes non-invasively.

From a methodological standpoint, the combined use of VOI-based MRI segmentation and radiomic feature extraction markedly enhanced sensitivity to treatment-associated tumor alterations compared with conventional imaging assessment [31]. While ultrasonography and standard MRI remain essential tools for anatomical characterization and longitudinal monitoring, radiomic processing mitigates inaccuracies related to irregular tumor geometry, asymmetric growth, and intratumoral heterogeneity [32]. This is particularly relevant in preclinical oncology, where tumors rarely conform to idealized geometric assumptions and where pharmacological effects may manifest primarily as microstructural rather than volumetric changes.

The histopathological findings provide biological context for the radiomic alterations observed in this study [27]. The examined tumors displayed pronounced intratumoral heterogeneity, characterized by regions of dense tumor cell proliferation, inflammatory infiltration, stromal remodeling, and extensive central necrosis. Such structural complexity is known to influence imaging texture patterns and may explain the radiomic changes detected in entropy, homogeneity, and run-length-based features. Necrotic regions and extracellular matrix accumulation can increase signal heterogeneity, while variations in cellular density and stromal composition may alter spatial gray-level distribution [29]. Therefore, the radiomic signatures identified in this study likely reflect underlying histological heterogeneity rather than differences in tumor size alone, supporting the concept that radiomics can act as a non-invasive surrogate of tumor microstructural characteristics.

Several limitations should be acknowledged. The relatively small size of the control group may have limited statistical power for selected comparisons, although this does not undermine the consistent and compound-specific radiomic patterns observed. In addition, radiomic features were not directly correlated with histopathological or molecular markers, representing an important direction for future validation. Nevertheless, the reproducibility of radiomic trends across imaging sequences and their concordance with biological patterns reported in the literature support the robustness and translational relevance of the present findings.

## 4. Materials and Methods

### 4.1. Materials

#### 4.1.1. Experimental Compounds and Reference Drugs

The investigational treatments comprised one synthetic cannabinoid and two phytocannabinoid-based formulations. The synthetic compound JWH-182 was acquired from Cayman Chemical (Ann Arbor, MI, USA). The phytocannabinoid products consisted of Cannabixir^®^ Medium dried flowers, 15.6% THC: <1% CBD- (NC1), and Cannabixir^®^ THC full extract, ∼20% THC- (NC2), both supplied by Cansativa GmbH (Mörfelden-Walldorf, Germany). As antineoplastic agent, paclitaxel, a semisynthetic taxane derivative with a certified purity of ≥98%, was purchased from Sigma-Aldrich (Darmstadt, Germany) and used according to standard preclinical oncology protocols.

#### 4.1.2. Cell Line and Culture Reagents

Tumor induction was performed using the murine mammary carcinoma cell line 4T1, obtained from the American Type Culture Collection (ATCC, Manassas, VA, USA). Cells were cultured under standard conditions in Dulbecco’s Modified Eagle Medium (DMEM) supplemented with fetal bovine serum (FBS) and penicillin–streptomycin. Cell harvesting and passaging were conducted using trypsin–EDTA. All cell culture reagents were sourced from Sigma-Aldrich (St. Louis, MO, USA).

#### 4.1.3. Animals and Housing Conditions

Female BALB/c mice (8–10 weeks of age, body weight 15–25 g) were used for all in vivo experiments. Animals were housed in the CEMEX animal research facility of the Grigore T. Popa University of Medicine and Pharmacy (Iași, Romania). Housing conditions were standardized, with controlled ambient temperature (20 ± 4 °C), relative humidity (50 ± 5%), and a 12 h light/dark cycle. Mice were maintained in individually ventilated cages (IVCs) and provided with ad libitum access to food and water.

#### 4.1.4. Anesthesia and Peri-Procedural Management

For all imaging procedures and experimental manipulations requiring immobilization, inhalational anesthesia was achieved using isoflurane (ISOFLUTEK^®^, 1000 mg/g inhalation vapor, liquid). The anesthetic agent was manufactured by Laboratorios KARIZOO, S.A. (Caldes de Montbui, Spain) and distributed in Romania by the authorized holder, Maravet SA (Baia Mare, Romania).

#### 4.1.5. Ethical Approval and Regulatory Compliance

All animal procedures were performed in strict accordance with European Directive 2010/63/EU on the protection of animals used for scientific purposes. The experimental protocol was reviewed and approved by the institutional Research Ethics Committee of the Grigore T. Popa University of Medicine and Pharmacy (approval no. 47/17.02.2021) and received authorization from the Romanian National Sanitary Veterinary and Food Safety Authority (approval no. 34/07.04.2021).

### 4.2. Methods

**Figure 9:**
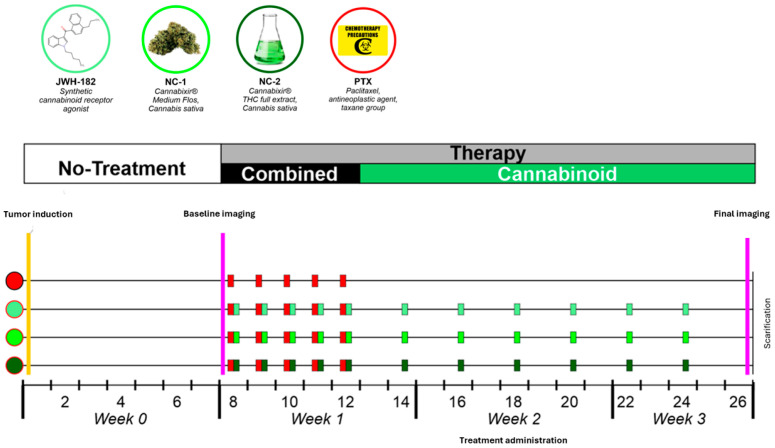
Summary of the study design.

The in vivo experimental protocol, including animal handling, tumor induction, treatment allocation, and drug administration, was conducted as previously described in detail in our published work [26]. Briefly, a longitudinal imaging-based design was employed to monitor tumor evolution in an orthotopic murine breast cancer model before and after cannabinoid administration. A summary of the study design is presented in Figure 9.

#### 4.2.1. Animal Allocation, Group Assignment and Tumor Induction

Naive female BALB/c mice were allocated to experimental groups prior to tumor implantation using a predefined randomization scheme. Animals were allocated to four treatment arms according to the administered compound: JWH-182 (*n* = 29), NC1 (*n* = 23), NC2 (*n* = 23), and a paclitaxel-only control group (*n* = 6). The numbers reported correspond to the animals that completed the imaging protocol and were included in the final imaging-based analyses. The animal workflow is presented in Table 2.

Further, orthotopic mammary tumors were generated using the murine 4T1 breast cancer cell line, by injecting the cell suspension subcutaneously into the sixth mammary fat pad of each mouse. Tumor development was monitored by palpation, and a solid, well-defined nodule adherent to adjacent tissues was consistently detectable within seven days following implantation.

#### 4.2.2. Treatment Schedule and Drug Administration

Following confirmation of tumor induction and acquisition of baseline imaging, pharmacological exposure was initiated. The intervention schedule consisted of an initial cannabinoid induction phase (day 8 post-implantation), a short concomitant exposure phase with paclitaxel (days 9–12), and a subsequent maintenance phase in which cannabinoids were administered alone on alternating days for 14 days.

All animals received paclitaxel by intraperitoneal injection at a fixed dose corresponding to 2 mg/kg. In the synthetic cannabinoid arm, JWH-182 was administered intraperitoneally using a saline-based vehicle supplemented with 1% polysorbate 80; dosing was standardized to 2.627 mg/kg, delivered in a volume normalized to body mass (1 mL per 100 g). Animals assigned to phytocannabinoid treatment received oral formulations by gavage, with NC1 administered at a higher mass-adjusted dose (75 mg/kg) and NC2 administered at a lower dose (12.72 mg/kg), reflecting differences in formulation and cannabinoid content. The motivation to choose the dosages is detailed in our previous work [25]. Control animals were exposed to paclitaxel under identical conditions and received vehicle alone in place of cannabinoids.

#### 4.2.3. Imaging Evaluation Using Ultrasonography (US), Magnetic Resonance Imaging (MRI) and Region of Interest (ROI) Processing

Baseline imaging was performed on day 8 after tumor implantation, before treatment initiation, while the final imaging assessment was performed on day 26. At both time points, tumor volume was assessed using US and MRI to enable direct comparison of conventional imaging metrics with radiomics-derived parameters.

Ultrasonographic evaluation of induced breast tumors was performed with the VisualSonics Vevo^®^ 2100 Imaging System (Fujifilm VisualSonics, Toronto, ON, Canada) ultrasound scanner, using the MS 400 high-frequency probe (Frequency 30 MHz, Power 100%, Gain 28 dB, Frame Rate 13, Depth 12 mm, Width 15.36 mm, Line Density High, Persistence Off, Sensitivity High). Isoflurane 3% was used for induction of anesthesia, which was then maintained by a continuous flow of an air/isoflurane 2% mixture. The subject was positioned on the examination table in the supine position and the fur was removed from the area to be examined using a standard hair trimmer. The average ultrasound examination time was 3–5 min/subject, during which time the general appearance of the tumor, anatomical relationships and dimensions in 3 planes (anteroposterior, transverse and craniocaudal) were assessed, with archiving of relevant images and measurements. After the examination, the animals were monitored until fully awakened from anesthesia and their well-being was assessed, after which they were transported to the housing room. The ultrasound evaluation procedure was applied to each subject three times during the experiment (day 8, day 15 and day 26). The MS400 provides high spatial resolution (reported axial resolution 40–50 μm and lateral resolution 80–100 μm), enabling detection of small structural changes in tumor morphology.

Acquisition of whole-body MRI images was achieved using 1T PET/MRI (nanoScan^®^) with a body mouse bed and 35 mm coil. Isoflurane 3% was used for induction of anesthesia, which was then maintained by a continuous flow of an air/isoflurane 2% mixture. Acquired sequences: axial T1 GRE using the following parameters: TR = 12.1 ms, TE = 3.3 ms, flip angle = 15°, number of slices = 80, slice thickness = 1 mm, field of view (FOV) = 35 × 36 mm, matrix = 320 × 144, spatial resolution = 0.109 × 0.25 mm^2^ and number of excitations (NEX) = 2; axial T2 FSE with the following parameters: TR = 17,286 ms, TE = 48.6 ms, echo train length (ETL) = 16, number of slices = 80, slice thickness = 1 mm, FOV = 34 × 35 mm, matrix = 150 × 128, spatial resolution = 0.227 × 0.273 mm^2^. The total examination time was ~15 min for each animal. Images were analyzed on an InterView Fusion Nucline post-processing station using dedicated Nucline software (Mediso nanoScan^®^ v.20), performing manual regions of interest (ROIs) of the tumor slices and tumor segmentation using the defined ROIs to obtain a volume of interest (VOI). A total of 39 morphometric and signal intensity features were calculated per VOI (detailed in Table 1). This was performed per animal, on both the T1 GRE and T2 FSE images at baseline and at the end of the experiment. Four radiomic datasets resulted for each subject. The relative ratio of post-treatment to baseline feature values was computed and used for statistical analysis to account for the repeated-measures design. Tumor segmentation was performed manually on each tumor-containing slice by a single experienced imaging operator using Mediso InterView Fusion/Nucline v1 software. Segmentation was based on visible tumor boundaries in both T1- and T2-weighted images, and the resulting slice-wise regions of interest were combined to generate a three-dimensional VOI. Intratumoral heterogeneity, including necrotic or edematous areas identifiable within the tumor mass, was included in the VOI because these components represent intrinsic biological characteristics relevant for radiomic analysis. Because segmentation was performed by a single blinded operator following a standardized procedure, formal inter-observer variability analysis was not conducted. Radiomic features were extracted using Mediso Nucline software (nanoScan^®^ v.20), which implements standard definitions of morphometric, first-order, and higher-order texture features derived from GLCM, GLRLM, and GLSZM matrices. Feature extraction was performed using the native voxel resolution of the acquired images and the default preprocessing parameters of the software. Intensity normalization and gray-level discretization were applied according to the software’s default preprocessing settings, ensuring consistency across all analyzed datasets.

#### 4.2.4. Antitumor Activity Assessment Using Tumor Doubling Time

Tumor growth dynamics were evaluated by calculating the tumor volume doubling time (DT), a parameter proposed by Steel et al. [29], commonly used to describe the rate of neoplastic expansion, where tumor growth was modeled assuming an exponential pattern, whereby the rate of increase is proportional to the existing tumor volume.

For MRI datasets in which tumors were segmented, volumetric values were extracted directly from the three-dimensional volume of interest (VOI) generated after manual delineation. In contrast, for ultrasonography and conventional (non-segmented) MRI assessments, tumor volume was estimated by approximating the lesion as an ellipsoid, using three orthogonal linear dimensions, anteroposterior (*AP*), transverse (*TS*), and craniocaudal (*CC*), according to the standard geometric formula $V=\frac{\pi }{6}\times AP\times TS\times CC$ [33]. To enable comparison of tumor growth dynamics across animals and imaging modalities, the tumor-specific growth rate (*SGR*) was calculated. As described by Mehrara et al. [32], *SGR* reflects the relative change in tumor volume per unit time and is derived using the logarithmic formulation $\;SGR=\frac{ln(V_{2}/V_{1})}{t_{2}-t_{1}}[\%/\mathrm{day}]$. In this equation, $V_{1}$ represents the tumor volume measured at the baseline imaging time point (day 8 after tumor implantation, prior to treatment initiation), while $V_{2}$ corresponds to the tumor volume measured at the final imaging assessment (day 26). The variables $t_{1}$ and $t_{2}$ denote the respective post-implantation time points. Tumor volume doubling time was subsequently calculated from the estimated *SGR* using the following logarithmic relationship [33]:$\;DT=\frac{ln(2)}{SGR}$.

#### 4.2.5. Histopathology Report

After experiments, animals were euthanized after prior anesthesia using isoflurane 2%. Primary tumors were collected at necropsy and fixed in 10% neutral-buffered formalin for histopathological evaluation. Tissues were trimmed according to standardized mouse sampling guidelines [34], processed using the Excelsior™ AS Tissue Processor (Epredia, Vista, CA, USA), and embedded in paraffin blocks. Paraffin blocks were sectioned at 4 µm using a semi-automatic microtome (CUT 5062, Slee Medical, Mainz, Germany), and sections were stained with hematoxylin and eosin (H&E). Slides were digitized at ×400 magnification using an Aperio AT2 DX slide scanner (Leica Biosystems Imaging, Vista, CA, USA). Histological evaluation focused on the structural characteristics of the primary tumors, including tumor architecture, cellular atypia, inflammatory infiltration, and the presence of necrotic or apoptotic areas.

#### 4.2.6. Statistical Analysis

Statistical evaluation of treatment effects was performed using the R statistical environment, version 4.5. Tumor growth dynamics were quantified by calculating tumor volume doubling time between two predefined imaging assessments: the baseline measurement obtained eight days after tumor implantation and the final measurement acquired on day 26. Prior to inferential analysis, data distribution was examined using the Shapiro–Wilk test to assess normality assumptions. As the distribution of doubling time values did not satisfy parametric criteria, non-parametric methods were employed. Overall differences among treatment groups were first evaluated using the Kruskal–Wallis test. When a significant group effect was detected, post hoc pairwise comparisons were conducted using the Wilcoxon rank-sum test for independent samples, with continuity correction applied. To control for inflation of type I errors associated with multiple testing, *p*-values from pairwise analyses were adjusted using the FDR correction.

Differences in the effects of the three drugs on the 39 radiomic features were tested via exact non-parametric permutation tests in conjunction with *t*-test statistics, using Python 3.10 and the SciPy package (version 1.11.3). Permutation tests are well-established for analyzing pairwise comparisons in small-sample biological datasets where parametric assumptions may not hold [35]. For each radiomic feature, pairwise comparisons were performed between the control group and each of the three treatment groups. Permutation tests were conducted by randomly shuffling the group labels across the entire dataset and calculating the *t*-test statistic for each permutation. This process was stochastically repeated 10,000 times to generate an empirical null distribution of the test statistic under the assumption of no group differences. Finally, to strictly address the multiplicity of testing 39 radiomic features, the resulting *p*-values were adjusted using the FDR procedure. The *p*-value for each pairwise comparison was then determined by calculating the proportion of permutations that resulted in a test statistic greater than or equal to the observed value. The relationship between the radiomic features within each group was determined using Pearson’s correlation coefficients. Permutation tests were employed to assess the statistical significance of these correlations, following a similar approach to that described above. For each pair of features, the data were permuted 10,000 times and the correlation coefficient was calculated for each permutation, generating a null distribution. The *p*-value was then computed as the proportion of permutations that yielded a correlation coefficient greater than or equal to the observed value in absolute terms. The permutation tests and associated *p*-values provided a robust and distribution-free approach for the evaluation of the statistical significance of the findings, accounting for the small sample sizes and potential violations of parametric assumptions.

## 5. Conclusions

MRI-based radiomics may represent a sensitive, non-invasive imaging approach capable of detecting treatment-associated tumor phenotypic alterations that are not captured by conventional ultrasonography or MRI-derived volumetric measurements. In this preclinical breast cancer model, radiomic analysis suggested compound-specific modulation of tumor architecture despite broadly comparable tumor growth kinetics across treatment groups. However, these findings should be interpreted cautiously considering the exploratory design of the study, the relatively small control cohort, and potential confounding effects related to differential tumor growth dynamics. Within these limitations, the present results indicate that radiomic analysis may provide complementary information to conventional imaging metrics when evaluating complex pharmacological exposures in preclinical oncology models. Further studies with larger and more balanced cohorts will be necessary to confirm these observations and to better define the role of radiomics as a pharmaco-imaging tool in anticancer drug development.

## Figures and Tables

**Table 1:** MRI radiomic variables calculated on tumor volumes in T1 and T2 images at the beginning and at the end of the experiment.

Primary	Higher Order		
Histogram	Gray-Level Co-occurrence Matrix (GLCM)	Gray-Level Run-Length Matrix (GLRLM)	Gray-Level Size-Zone Matrix (GLSZM)
Deviation	Homogeneity	Short-Run Emphasis	Short-Zone Emphasis
Mean	Correlation	Long-Run Emphasis	Long-Zone Emphasis
Max	Contrast	Low Gray-Level Run Emphasis	Low Gray-Level Zone Emphasis
Min	Coarseness	High Gray-Level Run Emphasis	High Gray-Level Zone Emphasis
Sum	Busyness	Short-Run Low Gray-Level Emphasis	Short-Zone Low Gray-Level Emphasis
Volume	Complexity	Short-Run High Gray-Level Emphasis	Short-Zone High Gray-Level Emphasis
Max. Diameter		Long-Run Low Gray-Level Emphasis	Long-Zone Low Gray-Level Emphasis
Entropy		Long-Run High Gray-Level Emphasis	Long-Zone High Gray-Level Emphasis
Size Variance		Gray-Level Non-Uniformity	Gray-Level Non-Uniformity
Intensity Variance		Run-Length Non-Uniformity	Zone Length Non-Uniformity
Kurtosis		Run Percentage	Zone Percentage

**Table 2:** Animal workflow throughout the study.

Animal Workflow	JWH-182	NC1	NC2	Control
Allocated	30	24	24	6
Imaged at baseline	30	24	24	6
Imaged at endpoint	30	24	24	6
Successfully segmented	29	23	23	6
Included in the final statistical analysis	29	23	23	6

## Data Availability

The raw data supporting the conclusion of this article will be made available by the authors, without undue reservation.
